# MitoTam-01 Trial: Mitochondrial Targeting as Plausible Approach to Cancer Therapy. Comment on Yap et al. Complex I Inhibitor of Oxidative Phosphorylation in Advanced Solid Tumors and Acute Myeloid Leukemia: Phase I Trials. *Nat. Med.* 2023, *29*, 115–126

**DOI:** 10.3390/cancers15184476

**Published:** 2023-09-08

**Authors:** Jiri Neuzil, Jakub Rohlena, Lukas Werner, Zuzana Bielcikova

**Affiliations:** 1 School of Pharmacy and Medical Science, Griffith University, Southport, Qld 4222, Australia; 2 Institute of Biotechnology, Czech Academy of Sciences, 252 50 Prague, Czech Republic; jakub.rohlena@ibt.cas.cz (J.R.); lukas.werner@ibt.cas.cz (L.W.); 3 Faculty of Science, Charles University, 128 00 Prague, Czech Republic; 4 First Faculty of Medicine, Charles University, 121 08 Prague, Czech Republic; zuzana.bielcikova@vfn.cz; 5 General University Hospital, Charles University, 128 08 Prague, Czech Republic

A recent paper published in *Nature Medicine* reported on the Phase I clinical trial of a mitochondria-targeting anti-cancer agent IACS-01059 in patients with acute myeloid leukemia (AML) and solid tumors [1]. Overall, 23 patients with solid tumors and 17 patients with AML were enrolled into the study. IACS-010759 is a small molecule (Figure 1) that was originally reported in 2018 as an agent that suppresses oxidative phosphorylation (OXPHOS) in chronic lymphocytic leukemia (CLL) cells by targeting mitochondrial respiratory complex I (CI), thereby promoting glycolysis [2]. Interestingly, the effect of IACS-010759 results in decreased ribonucleotide pool in CLL cells [2]. A follow-up paper reported that IACS-010759 binds to a specific site within CI with the ensuing blockage of reverse and forward electron flow [3]. Of note, the toxicity of IACS-010759 was reinforced by its combination with the glycolysis inhibitor 2-deoxyglucose, [2] the BH3 mimetic venetoclax, [4] or immune checkpoint inhibitors [5]. Recently, IACS-010759 was reported to reverse NOTCH1 signaling, which drives lymphoma [6], as well as being present in cancers with isocitrate dehydrogenase-1 mutations [7]. These findings, together with efficacy in a pre-clinical model of brain cancer and AML [8], resulted in the launch of the clinical trial of IACS-010759, as mentioned above [1].

The results of the IACS-010759 Phase 1 clinical trial [1] were critically assessed in a short opinion published in the same issue of *Nature Medicine* [9]. This report questions the results of the Phase I IACS-010759 trial, [1] in which the agent caused considerable toxicity in only one patient, with a solid tumor showing an objective response. The authors of the short opinion paper expressed doubts concerning targeting mitochondria as means of efficient tumor therapy, specifically stating that this particular clinical trial ‘faces failure’, and also stressing that several other clinical trials with mitochondria-targeting agents were unsuccessful. This is exemplified by the tricarboxylic acid cycle inhibitor CPI-613 (devimistat) which was highly promising in Phase I trial [10], but failed in later phases due to a lack of an anti-tumor effect. The authors of the article propose a ‘pause on mitochondria-targeting cancer therapies’ and the ‘thorough re-evaluation of the strategy’, as well as ‘to go from the bedside back to the bench’ [9]. They end their not-too-optimistic opinion by citing the statement of Hippocrates: ‘Primum non nocere’.

We purport that the pessimistic view of mitochondrial targeting as an anti-cancer strategy [9] does have a ‘silver lining’. We have focused on the design, synthesis, and testing of anti-cancer agents by tagging small molecules with the mitochondrial vector triphenylphoshonium (TPP^+^) that anchors biologically active agents at the interface between the inner mitochondrial membrane (IMM) and the mitochondrial matrix [11,12]. We have designed and tested several TPP^+^-tagged anti-cancer agents of which mitochondrially targeted tamoxifen (MitoTam) (Figure 1) appeared to be particularly intriguing based on experiments with tissue culture and mouse models of cancer, including its combination with immune checkpoint inhibitors [13,14]. MitoTam targets CI, binding within its Q-module, thereby blocking the entry of ubiquinone that intercepts electrons, resulting in increased level of reactive oxygen species [13]. MitoTam also, by virtue of its ‘intercalation’ into the IMM, causes the dissipation of the mitochondrial potential, leading to both apoptosis and necroptosis [14].

Based on research and pre-clinical results, we launched the Phase I/Ib trial of MitoTam with patients with untreatable, metastatic solid tumors, including some 10 types of cancer [15]. In Phase I, we treated 37 patients with MitoTam using the 3 + 3 strategy, until the maximum tolerated dose (MTD) was reached. In Phase Ib (38 patients), we evaluated the long-term toxicity of MitoTam applied using three regimens by differing the dose of MitoTam and frequency of its application. MitoTam was well tolerated in regimens 1 and 2, and afforded benefits to 78% patients in regiment 2 (3 mg/kg MitoTam applied once per week over 6 weeks), which is defined as stable disease or a (partial) response. Interestingly, and rather unexpectedly, the renal cell carcinoma (RCC) patients experienced the greatest benefit of all those with other diagnoses, with 83% RCC patients showing a stable disease or partial remission (MitoTam was applied for two or more therapeutic cycles), followed by a stable disease for up to three years after trial finalization. Linked to this, we found the highest accumulation of MitoTam in the kidney [14].

Notwithstanding the pessimistic outlook concerning mitochondria-targeted compounds, such as the above mentioned IACS-010759 and CPI-613, our research and the ensuing clinical tests show that there are mitochondria-targeted agents of imminent clinical use, which are epitomized by MitoTam. In this regard, we are now in the stage of planning the Phase 2 trial, hoping to give RCC patients an anti-cancer agent that targets mitochondria by means of a specific moiety that causes its accumulation in the vicinity of its molecular target. This would indicate that mitochondrial targeting, exemplified by the TPP^+^ tagging of small molecules with anti-cancer efficacy and molecular target within mitochondria (mitocans [16]), is a plausible strategy for efficient cancer therapy. We do agree with the authors of the article [9] that one needs to consider the type of cancer and the molecular mechanism by which mitochondria-targeted agents exert their activity. This is particularly important given the unprecedented plasticity of cancer cells that affects their metabolism and optimizes their energy generation means to comply with the unfavorable tumor environment or therapeutic challenges [17,18].

## Figures and Tables

**Figure 1:**
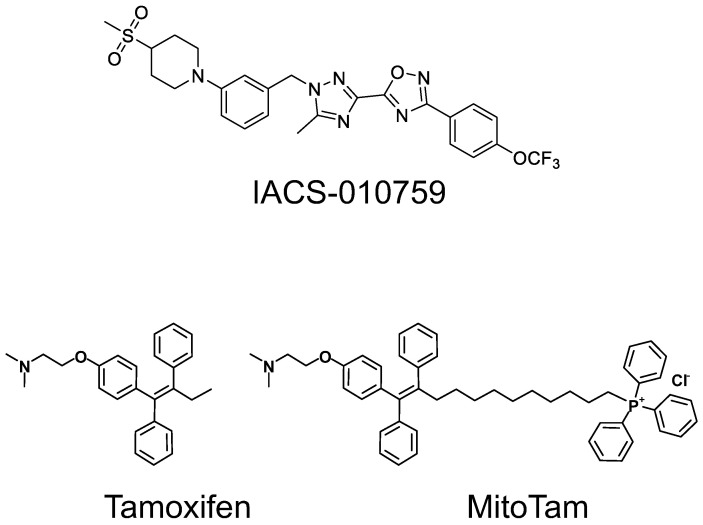
Structure of mitochondria-targeting anti-cancer agents.
